# The impact of dietary calcium and phosphorus on mitochondrial-linked gene expression in five tissues of laying hens

**DOI:** 10.1371/journal.pone.0270550

**Published:** 2022-06-24

**Authors:** Clara Dreyling, Martin Hasselmann

**Affiliations:** Department of Livestock Population Genomics, Institute of Animal Science, University of Hohenheim, Stuttgart, Germany; University of Life Sciences in Lublin, POLAND

## Abstract

Mitochondria and the energy metabolism are linked to both, the availability of Ca and P to provide the eukaryotic cell with energy. Both minerals are commonly used supplements in the feed of laying hens but little is known about the relationship between the feed content, energy metabolism and genetic background. In this study, we provide a large-scaled gene expression analysis of 31 mitochondrial and nuclear encoded genes in 80 laying hens in the context of dietary P and Ca concentrations. The setup included five tissues and gene expression was analysed under four different diets of recommended and reduced Ca and P concentrations. Our study shows, that mitochondrial gene expression is reacting to a reduction in P and that an imbalance of the nutrients has a higher impact than a combined reduction. The results suggest, that both strains (Lohmann Brown and Lohmann Selected Leghorn) react in a similar way to the changes and that a reduction of both nutrients might be possible without crucial influence on the animals’ health or gene expression.

## Introduction

Phosphorus (P) is an essential mineral for all living organisms which must be continuously supplied and is needed in poultry for growth, health and the energy metabolism [1]. The ability of poultry to degrade the natural present phytic-acid (InsP_6_) is limited [2, 3] and thus feed supplements derived from rock phosphate are added to maintain the P supply. The availability of rock phosphate is limited [4] and thus a reduction of its usage is of utmost interest.

Another important mineral essential for laying hens is calcium (Ca) [5] which is needed e.g. to form eggshells. Recent studies in broiler chickens have shown, that endogenous InsP_6_ degradation is reduced when mineral P and Ca are supplemented [6–8] and there is a strong interaction of P and Ca content regarding egg-shell quality and the number of produced eggs [9]. Many studies suggested, that the recommended dietary P content in the feed of laying hens might be too high, and can be reduced, without significant negative effects on performance and health of the animals [5, 10–12]. Hence, a better understanding of the effects of dietary P and Ca is necessary to implement these suggestions and adjust their dietary provision accordingly.

Both used strains (Lohmann Brown and Lohmann Selected Leghorn) are commercial important and selected for egg production [13–15]. Despite their similarities in egg production previous studies have shown differences between them concerning body weight, gene expression and phytate degradation in the context of the productive life span and changes in dietary Ca and P concentrations [10, 16, 17]. These results suggest differences in the strains reaction to dietary changes, which will be analysed in this work.

In this study we focus on mitochondrial gene expression since mitochondria are linked to P and Ca availability as well as to the animals’ fitness and energy metabolism. Mitochondria are the main energy producers of the cell through the process of oxidative phosphorylation (OXPHOS) [18] and this process depends on the availability [1] and is influenced by the concentration [19] of P. In addition, they play a major role in regulating Ca^2+^ homeostasis [20], which is an important factor in cell signalling since Ca^2+^ controls many cellular functions [21] including gene expression [22] and the regulation of OXPHOS [23].

In our experimental setup, we included all mitochondrial encoded OXPHOS subunits, as well as nuclear encoded ones such as *NDUFB6*, which is a subunit essential for the electron transport in complex I of the respiration chain [24] and *SOD2*, which detoxifies reactive oxygen species (ROS) produced during OXPHOS in mitochondria [25, 26]. In addition, we included nuclear genes which are part of the regulatory network, linking mitochondrial gene expression and biogenesis to external stimuli, such as *PGC1α* [27], nutrient sensitive factors such as *MTOR* as well as subunits of *AMPK*, which is part of the adaptive response to energy deficit [27]. Another important key player is *IGF-1α*, which has been described as participating in P transport [28], and a reduced IGF1 expression increase the effect of Ca deficiency on bone accretion in mice [29]. *IGF-1α* has also been linked to body weight in chickens [30], which makes it a promising gene in our experimental setup.

Preliminary studies mostly focus on physiological traits important for agricultural purposes, but also try to understand the mechanisms behind the effects of dietary P and Ca. In a study focusing on phytate degradation, transcellular mineral transporters, and mineral utilization of the same hens [10] no P-mediated effects were identified and a major question rising from this studies is, how the animals react to compensate the reduced amount of P and Ca. In this study, we focus on the expression of mitochondrial-linked and nutrient sensitive genes and test the following hypotheses:

1. Mitochondrial gene expression reacts to the reduction of P and Ca
2. The strains react differently to the changes in the diet composition
3. Genes regulating mitochondrial gene expression and biogenesis react to the dietary changes

Our study benefits from the already published analysis of phenotypic traits [10] as well from the known genetic background of the two strains [31]. Together with the possibility of a controlled environment during the experimental procedure it was possible to detect and analyse gene expression changes in the context of dietary adjustment.

## Material and methods

### Animals and experimental setup

The animal experiments were performed at the Agricultural Experiment Station of the University of Hohenheim, Germany. They were approved by the Regierungspräsidium Tübingen, Germany (Project no. HOH50/17TE) in accordance with the German Animal Welfare Legislation.

We used 80 laying hens: 40 Lohmann brown classic (LB) and 40 Lohmann LSL-classic (LSL) white leghorn hybrids contributed by Lohmann Tierzucht (Cuxhaven, Germany). The hens originated from an experiment addressing the utilization of phosphorus (P) and calcium (Ca) under different dietary conditions [10]. The experimental setup is described in detail in Sommerfeld, Omotoso, et al., 2020 [10] and will only be outlined briefly in the following.

The hens were reared together under standard conditions, with diets according to the requirements of each period, based on soy and corn meal. Ten father lines per strain were selected prior to the start of the experimental phase. After 27 weeks four hens per rooster were chosen and placed individually in metabolism units (1m × 1m × 1m) where the hens received specific diets for the following three weeks. Four different feed compositions were used (Table 1) and fed *ad libitum*, each group contained one hen per father line.

**Table 1:** P and Ca content (g/kg, dry mass) of the four diets. A table containing detailed information about the nutrients in the diets can be found in Sommerfeld et al., 2020.

Ingredient, g/kg	P+Ca+ (diet 1)	P-Ca- (diet2)	P+Ca- (diet3)	P-Ca+ (diet 4)
Calculated concentrations				
Total P	5.3	4.7	5.3	4.7
Ca	39.6	33.9	33.9	39.6
Analysed concentrations				
Total P	5.3	4.7	5.3	4.7
Ca	39.5	34.4	35.1	40.3

### Samples and RNA extraction

Samples of five tissues (breast muscle, ileum, duodenum, liver and ovary) were taken on four consecutive days with random distribution of the four diets in week 31. The animals were individually stunned with a gas mixture of 35% CO_2_, 35% N_2_, and 30% O_2_ and killed by decapitation at the Agricultural Experiment Station of the University of Hohenheim [10]. The samples were directly taken after slaughtering and were immediately placed on dry ice and stored at -80°C until RNA extraction.

RNA was extracted using TRIzol Reagent (Thermo Fisher scientific Inc., Massachusetts, USA) according to the manufacturers’ instructions with modifications described in Dreyling and Hasselmann 2022 [32]. Samples were dissolved in nuclease-free water, RNA concentration and quality in form of 260/280 and 260/230 ratios were measured using a NanoDrop 2000/2000c Spectrophotometer (Thermo Fisher scientific Inc., Massachusetts, USA). In addition to the provided NanoDrop values for all samples, a representative subset including samples of different quality and quantity was measured on a Qubit 4 (Thermo Fisher scientific Inc., Massachusetts, USA) using the Qubit RNA IQ Assay Kit (Thermo Fisher scientific Inc., Massachusetts, USA). The samples were stored at -80°C until further processing.

### Real time PCR

The primer design and assay evaluation are already published in Dreyling and Hasselmann (2022) [32], and were not repeated specific for this experiment. The experimental procedure is identical and only described in brief in the following. In this study, 28 candidate genes were used, whereas now five additional genes have been integrated into the final set of primers: *ATPF0*, *ND2*, *ND3*, *PRKAB1*, and *PRKAG3*. A list with the gene names and their abbreviations used in this study can be found in Table 2.The used primer set including product size, primer efficiency and accession numbers of the reference sequences can be found in S1 Table. In addition, melting and standard curves are provided which were produced the same way as described in Dreyling and Hasselmann (2022) [32].

**Table 2:** Genes used in this study with abbreviations and genome in which they are encoded.

Abbreviation	Genome	Gene
*ACTB*	Nuclear	Actin beta
*ATP6*, *ATP8*, *ATP5F1*, *ATPF0*	MitochondrialNuclear	ATP-synthase F_0_ subunits
*COX1*, *COX2*, *COX3*, *COXC6*, *COX5A*	MitochondrialNuclear	Cytochrome oxidase subunits
*CytB*	Mitochondrial	Cytochrome b
*GAPDH*	Nuclear	Glycerinaldehyd-3-phosphat-Dehydrogenase
*IGF-1α*	Nuclear	Insulin-like growth factor 1α
*MTOR*	Nuclear	Mechanistic target of rapamycin
*ND1-ND4*, *ND4L*, *ND5*, *ND6*, *NDUFB6*	MitochondrialNuclear	NADH:ubiquinone oxidoreductase subunits
*PGC1α*	Nuclear	Peroxisome proliferator-activated receptor gamma coactivator 1-α
*PPIA*	Nuclear	Peptidyl-prolyl cis-trans isomerase A
*AMPK (PRKAA1*, *PRKAA2*, *PRKAB1*, *PRKAB2*, *PRKAG2*, *PRKAG3)*	Nuclear	AMP-activated protein kinase and its α1, α2, β1, β2, γ1, and γ2 subunits
*SDHA*, *SDHB*	Nuclear	Succinate dehydrogenase complex subunits
*SOD2*	Nuclear	Superoxide dismutase
*UQCRC1*, *UQCRC2*	Nuclear	Cytochrome b-c1 complex subunits 1 and 2

All analyses were performed on a Biomark HD system (Fluidigm Corporation, San Francisco, USA), following the protocols for gene expression analysis using five 96.96 IFCs using the Delta Gene Assays protocol with the manufacturers standard protocol for fast PCR and melting curve as described in S2 Table. The protocol includes a DNase digestion prior to the reverse transcription and a pre-amplification with multiplexed primers followed by an Exonuclease I treatment. Pre-amplification bias was already described and discussed in Dreyling and Hasselmann (2022) [32]. All qPCRs were performed in duplicates and the samples were placed randomly on the chips, only grouped by individual to avoid any bias of sample arrangement. An internal control and a negative control (throughout all preparation steps) were included to detect variance between the runs and potential contamination. In each well of the IFC 2.25μl of the diluted Exo I digested sample were added, resulting in 3.015nl in each reaction chamber. Information about the primer-pairs and thermal cycling conditions can be found in Table A-C in S1 File.

### Data preparation

#### Quality control

For data evaluation and quality control the Fluidigm Real-Time PCR analysis software was used. Only Cq-values from reactions with logarithmic increase of fluorescence and specific melting points were used for the following analyses. After the automatic quality check of the software, the results were evaluated by eye and revised manually if necessary. The quality threshold was set to 0.65 and the peak ratio threshold to 0.8.

The results of the internal control were checked to detect possible variation due to technical issues.

#### Reference gene evaluation

We used *PPIA* and *ACTB* as reference genes for normalization. Our previous evaluation already showed that *GAPDH* is strongly influenced by tissue and thus we included it as candidate gene in our study. To verify that *GAPDH* shows high variance between tissues in this experiment as well, we performed a reference gene evaluation using Normfinder [33]. As input one individual of each strain and diet was used, including all five tissues per individual to cover all our variables of interest. Normalization was tested for tissue type and diet.

#### Calculating relative gene expression

Means of duplicates were calculated of all samples with two successful runs. For samples that only had one successful duplicate this run was used. Gene expression relative to the reference genes was calculated using the Pfaffl-method [34] as optimized for multiple reference genes [35, 36]:

$$rel.gene\;expression=\frac{{RQ}_{GOI}}{geomean[{RQ}_{refs}]}$$

Where *RQ* = *E*^Δ*ct*^ and $E=(\frac{primer\;efficiency\;\%}{100})+1$.

Δct was calculated as the difference between the average cycle threshold (ct) of the internal control to the ct of the corresponding sample. E refers to the converted primer efficiency and GOI to gene of interest. RQ are relative quantity values calculated using E and Δct. *PPIA* and *ACTB* were used as reference genes (refs). The data was log2 transformed prior to statistical analysis.

### Statistical analysis

Linear mixed models were implemented and adjusted to each of the genes.


Y∼strain+diet+tissue+tissue*strain*diet+tissue*strain+tissue*diet+strain+diet+individual+father+ε

Where Y is relative gene expression, ε is the residual error; strain, diet and tissue are fixed effects, with individual and father as random effects. All modelling was performed in R (R Core Team 2019, Version 3.6.1) using the *lmerTest* [37]. A three factorial analysis of variance (anova) was performed to evaluate the influence of fixed effects and pairwise tukey *posthoc* tests (package *emmeans* [38]) were performed to detect differences between strain, diet and tissues in various combinations. The output of the model is estimated marginal means (emmeans), which are used for statistical analyses throughout the whole study. The fulfilling of normal distribution and the homogeneity of variance were evaluated using QQ and residual plots. Outliers were removed for each dataset using the interquartile range, except for *SDHA*, *PRKAA2*, *PRKAB2* and *GAPDH* because for these datasets a removal of outliers would have included too many samples to perform a proper analysis.

## Results

After the quality filtering, we received a dataset of 13,487 ct values from 35 Genes and all 400 samples. From 184 samples we obtained high-quality ct values for all genes, whereas for the remaining samples at least one gene was missing. In the final analyses all samples were included since no relationship between sample quality and the successful run of all genes was recognizable. Tables containing concentrations and quality of the RNA extracts can be found in S3 File.

The calculated Δct values ranged from -6.49 (min. for *PRKAA2*) to 13.38 (max for *PRKAG3*).

The statistical model revealed, that no included gene was significantly influenced by the strain, two genes were influenced by diet and all genes by the tissue. The most frequent interaction was between strain:tissue (6), followed by strain:diet and strain:tissue:diet (1) while there was no significant interaction of tissue:diet. The results of the tests derived from the statistical models for all genes and factors can be found in S1 Table. Sample numbers per gene, strain, tissue and diet can be found in S2 and S3 Tables.

### Strain differences

**Fig 1:**
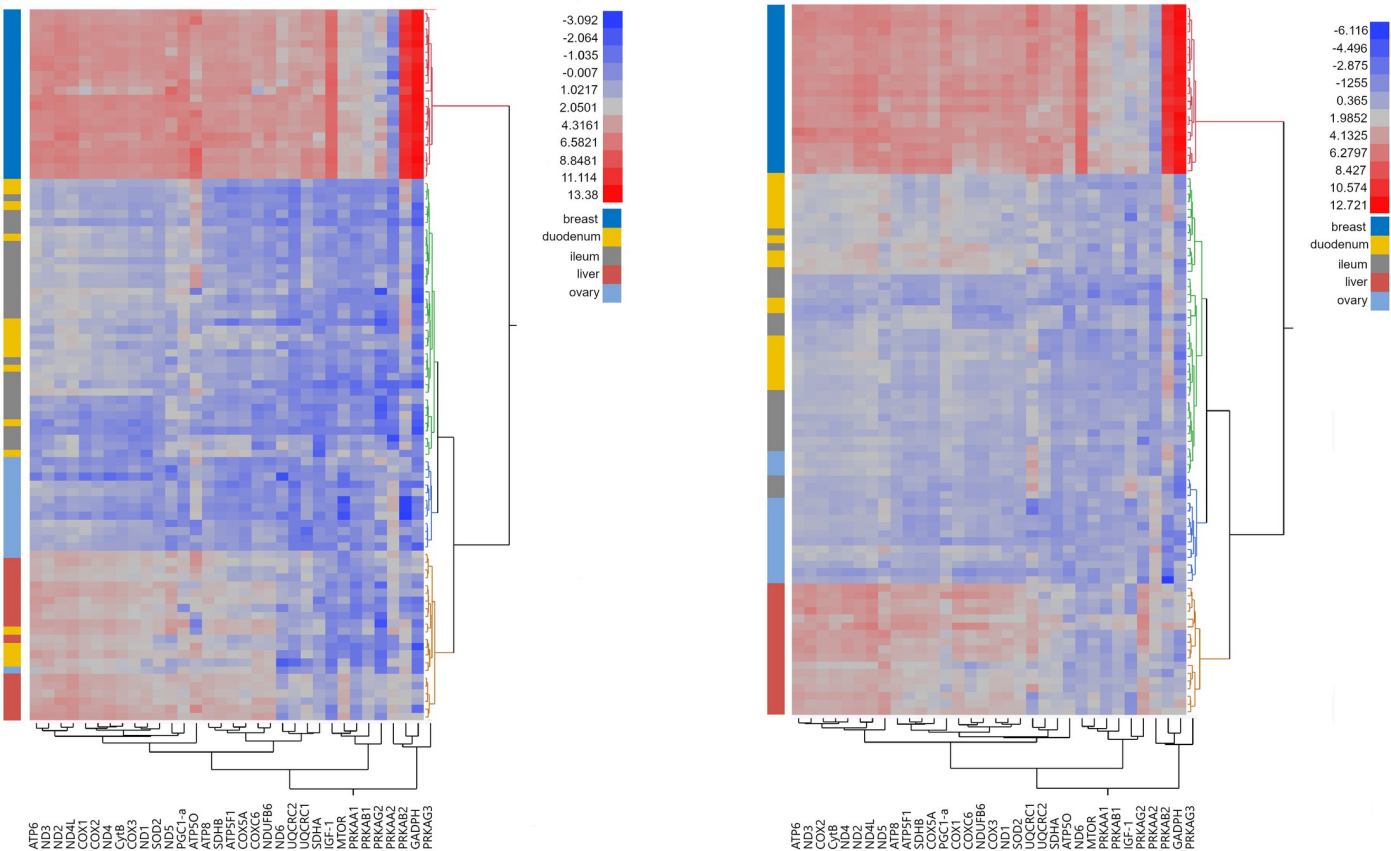
Heat maps of two-way hierarchical cluster analysis for individuals of the LB (left) and LSL (right) strain using Ward’s minimum variance method [40], the number clusters was estimated using the cubic clustering criterion [39].

The two-way hierarchical clustering analyses revealed a different number of clusters within each strain of laying hens. Two clusters were calculated by the cubic clustering criterion [39] for both strains (Fig 1). The analysis was performed on 92 samples of each strain, including 19 to 25 samples per diet per strain and 14 to 24 samples per tissue and strain. In general, both strains showed differentiation different tissue types, especially breast and liver, with more pronounced tissue specific expression pattern in the LSL strain. Consistent for both strains, a strikingly high up-regulation of three subunits of AMPK (*PRKAA2*, *PRKAB2* and *PRKAG3*) and *GAPDH* are found within breast tissue. The number of clusters was two in the LB and four in the LSL strain, which reflects the clearer separation of tissue types in the LSL strain.

**Fig 2:**
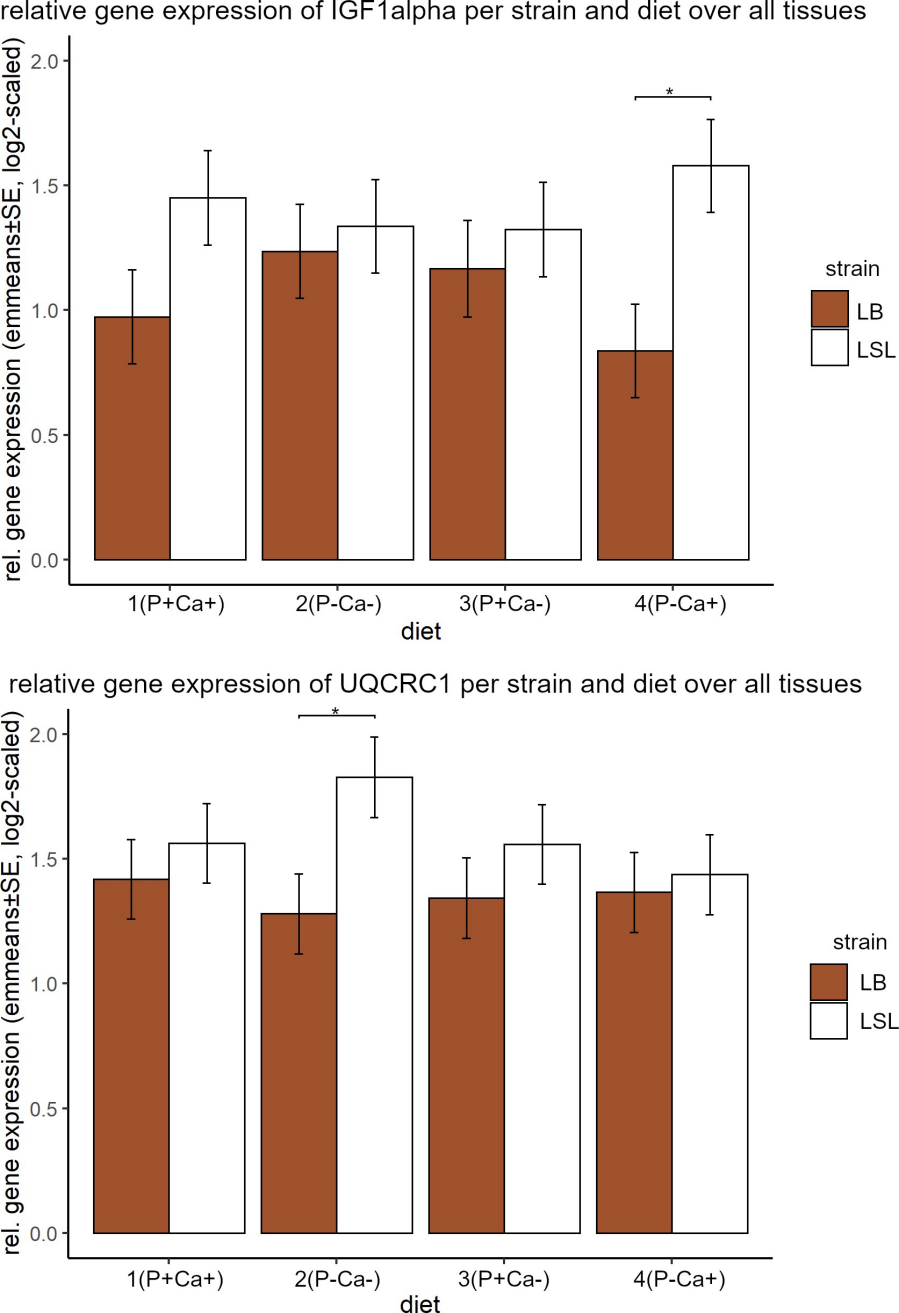
Relative gene expression of *IGF-1α* and *UQCRC1* of both strains for all diets. Shown are emmeans and standard errors estimated by the statistical model over all tissues. Statistical significance was declared when p<0.05.

Using our statistical linear mixed model, we tested for the overall impacts of strain, tissue and diet on gene expression. We observed no difference between the two strains for all genes under all four nutritional conditions, except for *IGF-1α* and *UQCRC1* (Fig 2). Both genes were showing higher gene expression in the LSL strain for all four diets, with a significant difference under low P and high Ca (diet 4) for *IGF-1α* (p = 0.0076), and low Ca and low P conditions (diet 2) for *UQCRC1* (p = 0.0214).

**Fig 3:**
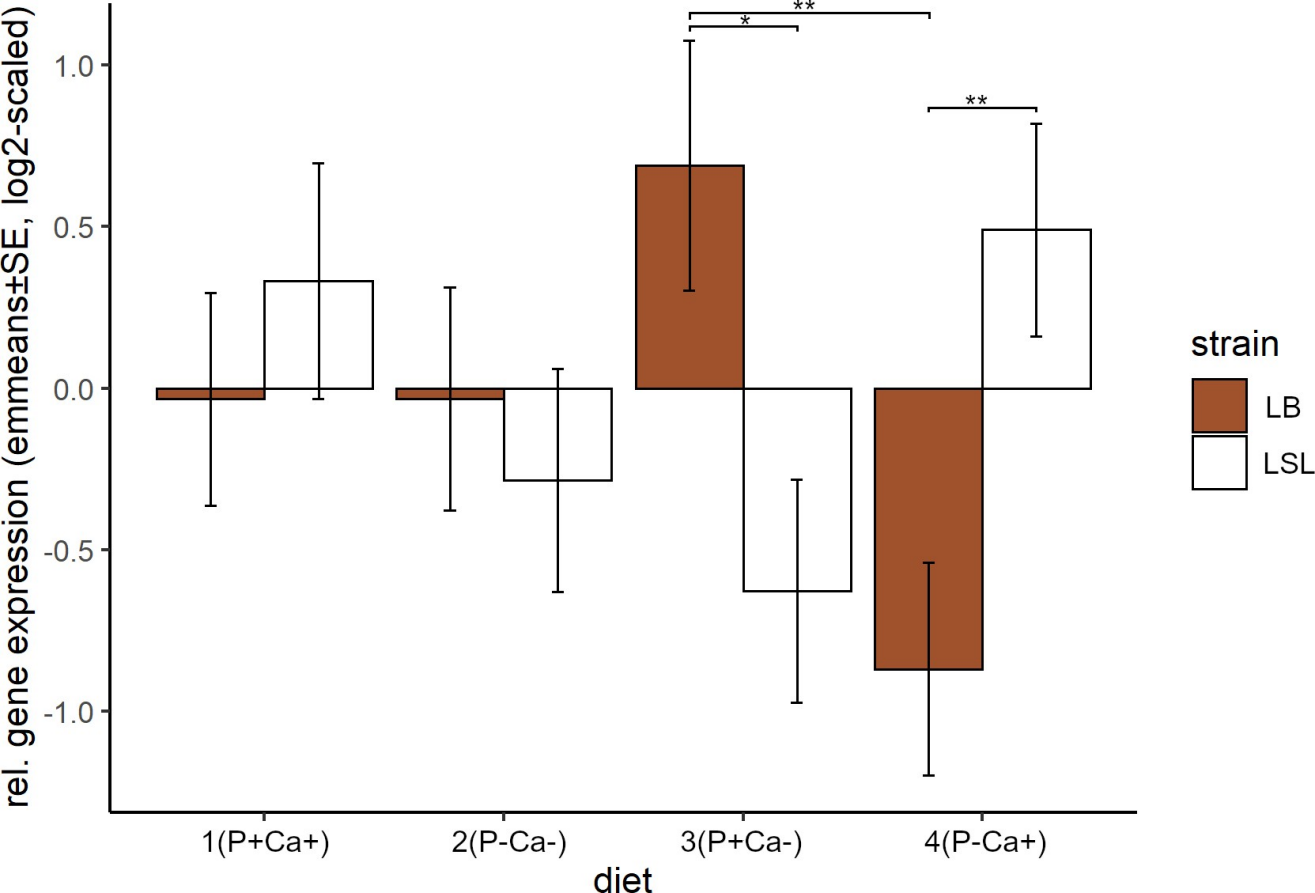
Relative gene expression of *IGF-1α* per strain and diet in ovary tissue. Shown are emmeans and standard errors estimated by the statistical model over all tissues. Statistical significance was declared when p<0.05.

For *IGF-1α* these strain differences are most pronounced in three tissues: breast (higher in LB hens under low P conditions (p = 0.0227 for P-Ca- and p = 0.0415 for P-Ca+)), liver (always higher in the LSL strain, p = 0.003 for P+Ca+, p = 0.0001 for P-Ca- and p<0.0001 for P+Ca- and P-Ca+) and ovary tissue with contrasting pattern among the diets under P+Ca- (p = 0.0116) and P-Ca+ (p = 0.0037) (Fig 3). In ovary tissue, the significant decrease of gene expression in the LB strain under P-Ca+ is consistent with the overall trend (Fig 5) while the increase in the LSL strain does not follow this pattern.

### Differences between the diets

**Fig 4:**
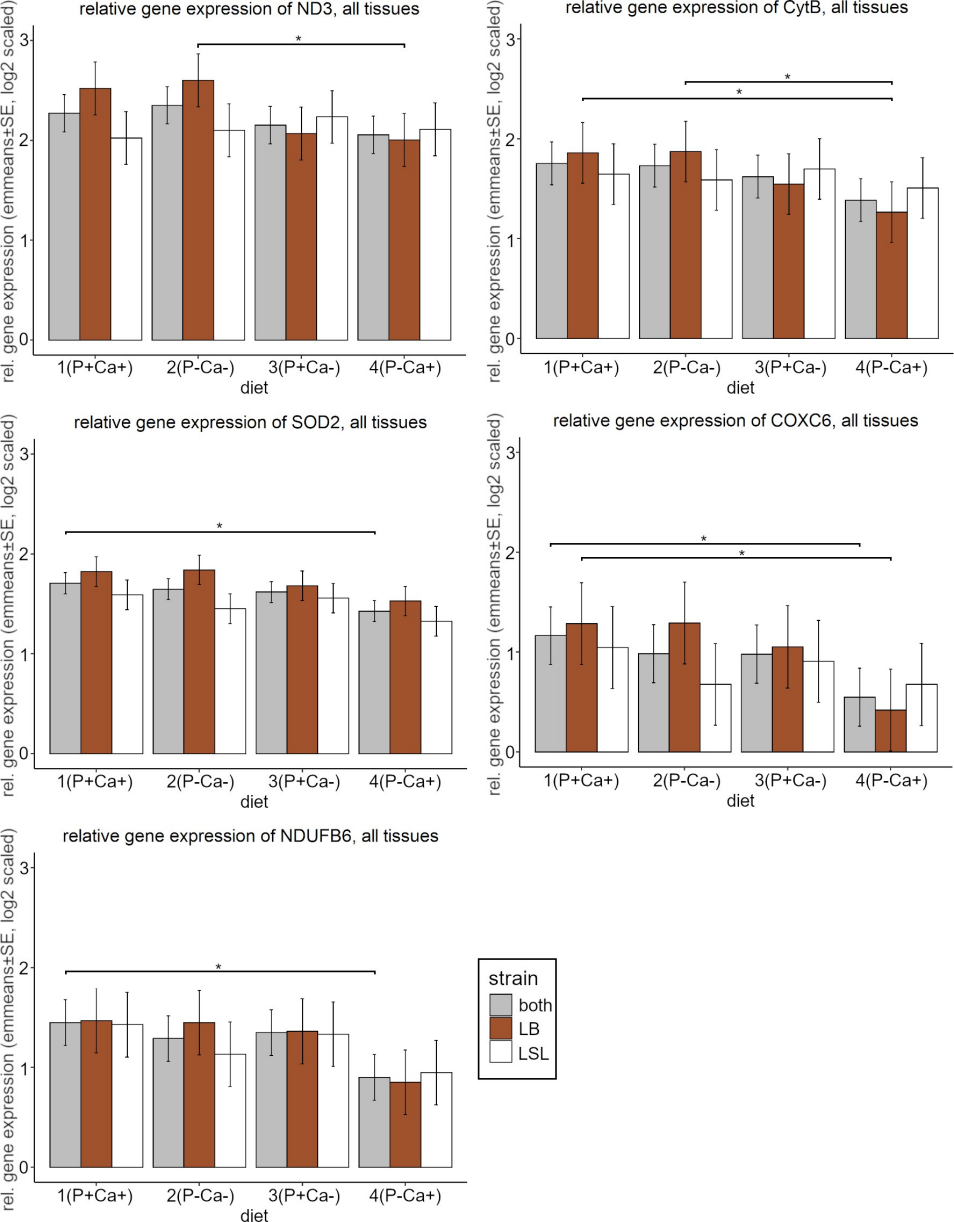
Relative gene expression of *ND3*, *CytB*, *SOD2*, *COXC6* and *NDUFB6* for both, the LB and the LSL strain for all diets. Shown are emmeans and standard errors estimated by the statistical model over all tissues. Statistical significance was declared when p < 0.05.

The overall gene expression (mean of all genes, tissues and strains per diet) was lowest under low P and high Ca conditions; however, the differences between the diets were not significant. The statistical model revealed, that the expression of only two genes (*SOD2* and *NDUFB6*) was affected by the diet. Nevertheless, with pairwise comparisons of the diets, more genes showed significant gene expression differences: *ND3*, *CytB*, *SOD2*, *COXC6*, and *NDUFB6*. For all these genes, the expression was lowest under low P and high Ca conditions. The expression of *SOD2*, *COXC6* and *NDUFB6* was significantly higher under high P compared to low P under high Ca conditions (Fig 4) for both strains analysed together. In addition, the LB strain showed expression differences in *CytB*, where the expression was higher under high P and Ca levels as well as under low P and Ca levels compared to diet 4 (P- Ca+).

In addition to the already described differences between the strains in the expression of *IGF-1α*, the differences between P+Ca- and P-Ca+ in ovary tissue were significant in the LB strain (p = 0.0062) and strong but not significant in the LSL strain (p = 0.0563) (Fig 3). Additionally, the expression in the LB strain was significantly higher in liver tissue under P-Ca- compared to P-Ca+ (p = 0.0088)

### Tissue differences

**Fig 5:**
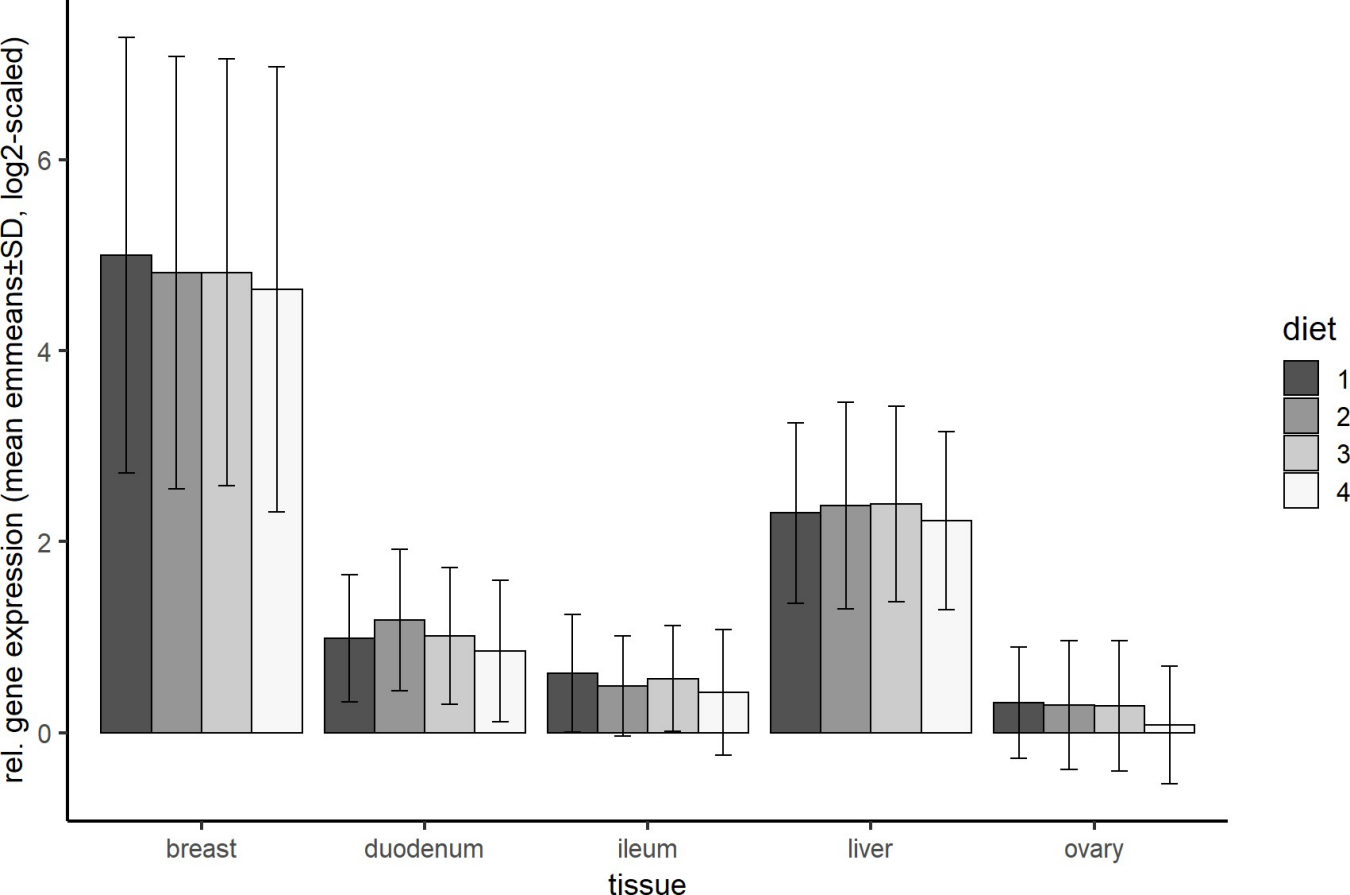
Relative gene expression in five tissues and diets. Shown are means and standard derivations of the emmeans of all genes calculated by the statistical model.

As already discussed in the context of time dependent gene expression [32], the tissue had the strongest influence in our experimental setup, influencing the expression of all included genes. The gene expression was significantly highest for all genes in breast muscle compared to the remaining four tissues (p<0.001 for all except *PRKAA1* where p = 0.048 when breast compared to liver), except for *IGF-1α* and *PRKAG2*, where there was no difference compared to liver tissue. The expression was lowest in all tissues when fed diet 4 (P-Ca+) (Fig 5). The already described diet-dependent gene expression changes in *SOD2* and *NDUFB6* were only observed in breast muscle tissue, where the expression was significantly lower under P-Ca+ than under P+Ca+ levels (for more details see S3 Table). In *GAPDH* highly significant differences between all pairwise tissue comparisons were observed (p<0.0001), which supports the decision to abandon it as a reference gene in our study.

## Discussion

In our experimental setup we were able to analyse a vast number of mitochondrial and nutritional linked genes in the context of dietary changes in P and Ca contents. We hypothesized, that one compensatory mechanism of changes in the diets is the adaption of mitochondrial gene expression, since it is directly linked to the availability of P and the fitness of the individuals.

### Differences between the strains

For none of our candidate genes, the gene expression differed between the strains, and only two genes (*IGF-1α* and *UQCRC1*) showed different gene expression in specific diets (under low P concentration). These observations indicate, that both strains react to the changes in dietary P and Ca content the same way and also in the hierarchical clustering analyses, the pattern of both strains was similar. A genome wide gene expression analysis comparing the same strains as included in this work identified genes related to the GO-cluster of phosphorous metabolism (GO-IDs: GO:006468, GO:0006793, GO:0006796, GO:0016310) to be down regulated in hens of the LSL strain [14] and Sommerfeld et al., 2020 [10] identified two sodium/phosphate co transporters to be higher expressed in the LSL strain using the same individuals as in this work. These results indicate, that there are differences between the strains related to P metabolism, however the genes included in this work showed no general differences between the two strains. It must also be noted, that Sommerfeld *et al*. 2020 [10] identified no interaction of strain and diet on the included genes, which supports the hypothesis that the reduction of P and Ca content in the used diets was not sufficient to improve the expression levels of our gene targets significantly. Even if we were able to show differences in gene expression between strains and diets in some genes in our study, the majority of the included genes showed no reaction to the dietary changes. A significant change in the mineral concentrations with an detrimental effect on the whole animal might lead to stronger effects on the animals as described in this work or by Sommefeld *et al*. 2020 [10].

### Mitochondrial gene expression in the context of dietary changes

Since both nutrients are linked to the mitochondrial energy-metabolism [1, 23] we suggested an adaption of mitochondrial gene expression as a compensatory reaction to the changing amount of the minerals. Our data revealed, that most genes showed no significant difference in gene expression according to the changes in the diet. However, the gene expression was lowest under P-Ca+ conditions, while the reduction of both minerals or only Ca had a smaller effect on gene expression. These results suggest, that the effect of reduced P concentrations is stronger, when there is an imbalance of the proportion, especially under low P.

Four of the five genes showing significant differences were part of the electron respiratory chain, representing complexes I, III, and IV and the ROS detoxifying gene *SOD2*. The gene expression was significantly lower under P-Ca+ compared to P+Ca+ in genes representing subunits from Complex I and IV of the respiration chain and the ROS detoxifying gene *SOD2*. The same observation was made for *CytB* in the LB strain (Fig 4). This observation suggests that the reduced availability of P impacts distinct parts of the respiratory system, resulting in reduced gene expression. Since it is known, that the expression of whole complexes can be regulated by the expression of individual subunits [41, 42], the reduced expression of single subunits might regulate the amount of the whole complex. Additionally, previous studies showed pattern of co-expression of the OXPHOS complexes [43], which is also shown in our analysis under low P and high Ca conditions.

A general reduction of assembled OXPHOS complexes might be the result of the observed expression pattern. The potential reduced production of ROS resulting from diminished OXPHOS activity leads to a reduced need of SOD2. SOD2 expression has been linked to impact immunity against bacterial infections in zebrafish [44] and ROS are known to play a role in the reaction to inflammatory disease [45–47]. Thus, the differences in SOD2 expression in laying hens might indicate differences in resistance to infections as well. In addition, an increase in the production of ROS in the mitochondrion is linked to the process of ageing in many species [48] and the increase of *SOD2*-expression protects the mitochondrion from damage, which would otherwise lead to the death of the cell (as stated in Santos et al., 2018 [48], Yin et al., 2018 [49] and cited references within). Regarding the missing of differences in gene expression between the other treatments suggests that a reduction of P alone is more crucial than a reduction of P and Ca or Ca.

### Gene expression differs between different tissue types

As described in the context of life span [32] the gene expression in breast muscle was significantly higher than in all other tissues, followed by liver tissue. Studies in humans and chimpanzees have shown, that the differences in gene expression are higher between tissues than between species and suggests to include different tissue types in functional genomics studies [50]. In liver, the high rates of gene expression might be explainable by the high amount of functions of the tissue, ranging from the conversion of glucose into glycogen, the filtration of blood to the production of cholesterol [51]. In the duodenum and ileum the mucosa and microbiome are important participants in the process of digestion and uptake of nutrients [52], which might explain the lower rates in the tissue itself. However, the high activity in breast muscle tissue is surprising, especially since the process of growing was already finished during sampling and the purpose of the strains focuses on egg laying instead of meat production. In general, there are not many studies comparing gene expression rates between tissues since most studies focus on differences between different treatments, diseases or developmental states.

### *IGF-1α* in the context of nutritional changes

We observed most differences in gene expression between strains and diets in the growth factor *IGF-1α*. This gene plays a major role in a variety of tissues and functions, e.g. metabolic homeostasis [53, 54], and growth [55, 56] and nutrition has been identified as a key factor of IGF1 regulation in humans [57, 58]. This sensitivity might be the reason of the significant differences in expression between the diets observed in this study, especially since malnutrition is known to reduce circulating IGF1 in mice [59]. Additionally, malnutrition has been linked to decreasing IGF1 expression in liver tissue [60], which is reflected by the significantly lower expression under P-Ca+ compared to P-Ca- in liver tissue of the LB strain in this study. This observation also leads to the conclusion, that an imbalance of the minerals is detrimental compared to the reduction of both minerals, and is thus a form of malnutrition. An interaction of P and Ca content have also been shown in the context od egg-shell quality and quantity of eggs [9]. Simultaneously the gene expression is significantly lower under P-Ca+ conditions in the LB strain, which is another indication of differences in the reaction to the reduction of Ca in both strains. The most prominent difference between the strains was the contrary expression of the strains under P+Ca- and P-Ca+ conditions. Even if it is long known, that IGF-1α plays a role in avian ovaries [61], we could not observe any changes in egg weight between the strains matching the change in expression pattern [10].

### Expression changes in nutrient sensitive and mitochondrial regulatory genes

We included nutrient sensitive genes such as *MTOR* and *AMPK* and the nutrient sensitive mitochondrial regulator *PGC1α* in our study and the missing reaction to our dietary changes is striking. All of these genes have been analysed in the context of changes during the productive life span of laying hens [32] using the same technical approach, which makes it rather unlikely, that our setup is failing in detecting expression changes. The same hens have been analysed in the context of performance traits such as body weight, feed intake, average egg weight and P/Ca efficiency, where no diet specific changes could be observed [10]. In accordance to other studies [5, 11, 12] the authors conclude, that a 20% reduction of P is not affecting the animals and thus, the recommended concentrations in the feed of these animals might be too high. The missing effect on genes that are part of the mitochondrial regulatory network supports this hypothesis.

## Conclusion

We performed a large-scaled analysis of mitochondria-linked gene expression in laying hens in the context of P and Ca content in the diets. Our study revealed interesting differences of gene expression of subunits covering most OXPHOS complexes under low P and normal Ca concentrations in the diets. Together with the decrease in the expression of the ROS detoxifying gene *SOD2*, an interesting part of the regulation of mitochondrial gene expression has been revealed. In addition, the effects on the growth factor IGF-1α showed that the reduction of P in the diet has an effect on the mitochondrial regulatory network as well. We also observed that an imbalance of both minerals seems to have greater influence of gene expression than the reduction of both nutrients, especially under low P conditions.

## Supporting information

S1 File Information about primer pairs and cycling conditions. (DOCX)

S2 File Standard and melting curves of the primers for ATP50, ND2, ND3 and PRKAG3. (XLSX)

S3 File NanoDrop measuremnts of all extracted samples. Samples that were used in the hierarchical cluster analysis and for that all genes run successful are marked.
(XLSX)

S1 Table Significant influence of strain, diet, tissue and all possible interactions on gene expression per gene. p-values from the three-factorial anova obtained from the linear mixed model. Statistical significance was declared when p < 0.05.
(DOCX)

S2 Table Number of samples per gene and tissue after the removal of outliers used to calculate emmeans from the statistical model. (DOCX)

S3 Table Number of samples per gene, tissue and diet after the removal of outliers used to calculate emmeans from the statistical model. (DOCX)

## References

[1] Elser JJ. Phosphorus: A limiting nutrient for humanity? Curr Opin Biotechnol. 2012;23(6):833–8. doi: 10.1016/j.copbio.2012.03.001 22465489

[2] Eeckhout W, De Paepe M. Total phosphorus, phytate-phosphorus and phytase activity in plant feedstuffs. Anim Feed Sci Technol. 1994;47(1–2):19–29.

[3] Rodehutscord M, Rückert C, Maurer HP, Schenkel H, Schipprack W, Bach Knudsen KE, et al. Variation in chemical composition and physical characteristics of cereal grains from different genotypes. Arch Anim Nutr. 2016;70(2):87–107. doi: 10.1080/1745039X.2015.1133111 26829392

[4] Cordell D, Drangert JO, White S. The story of phosphorus: Global food security and food for thought. Glob Environ Chang. 2009;19(2):292–305.

[5] Ahmadi H, Rodehutscord M. A meta-analysis of responses to dietary nonphytate phosphorus and phytase in laying hens. Poult Sci. 2012 Aug 1;91(8):2072–8. doi: 10.3382/ps.2012-02193 22802206

[6] Sommerfeld V, Schollenberger M, Kühn I, Rodehutscord M. Interactive effects of phosphorus, calcium, and phytase supplements on products of phytate degradation in the digestive tract of broiler chickens. Poult Sci [Internet]. 2018 Apr 1 [cited 2021 May 17];97(4):1177–88. Available from: https://pubmed.ncbi.nlm.nih.gov/29325118/ doi: 10.3382/ps/pex404 29325118 PMC5914422

[7] Shastak Y, Zeller E, Witzig M, Schollenberger M, Rodehutscord M. Effects of the composition of the basal diet on the evaluation of mineral phosphorus sources and interactions with phytate hydrolysis in broilers. Poult Sci [Internet]. 2014 [cited 2021 May 17];93(10):2548–59. Available from: https://pubmed.ncbi.nlm.nih.gov/25085939/ doi: 10.3382/ps.2014-03961 25085939

[8] Zeller E, Schollenberger M, Witzig M, Shastak Y, Kühn I, Hoelzle LE, et al. Interactions between supplemented mineral phosphorus and phytase on phytate hydrolysis and inositol phosphates in the small intestine of broilers. Poult Sci [Internet]. 2015 Feb 18 [cited 2021 May 17];94(5):1018–29. Available from: https://pubmed.ncbi.nlm.nih.gov/25810408/ doi: 10.3382/ps/pev087 25810408

[9] Härtel H. Evaluation of the dietary interaction of calcium and phosphorus in the high producing laying hen. Br Poult Sci. 1990;31(3):473–94. doi: 10.1080/00071669008417280 2245345

[10] Sommerfeld V, Omotoso AO, Oster M, Reyer H, Camarinha-Silva A, Hasselmann M, et al. Phytate degradation, transcellular mineral transporters, and mineral utilization by two strains of laying hens as affected by dietary phosphorus and calcium. Animals. 2020;10(10):1736. 10.3390/ani10101736 PMC7598718 32987788

[11] Jing M, Zhao S, Rogiewicz A, Slominski BA, House JD. Assessment of the minimal available phosphorus needs of pullets during the pre-laying period. Poult Sci. 2018 Feb 1;97(2):557–67. doi: 10.3382/ps/pex313 29077938

[12] Pongmanee K, Kühn I, Korver DR. Effects of phytase supplementation on eggshell and bone quality, and phosphorus and calcium digestibility in laying hens from 25 to 37 wk of age. Poult Sci. 2020 May 1;99(5):2595–607. doi: 10.1016/j.psj.2019.12.051 32359595 PMC7597456

[13] Singh R, Cheng KM, Silversides FG. Production performance and egg quality of four strains of laying hens kept in conventional cages and floor pens. Poult Sci [Internet]. 2009;88(2):256–64. Available from: doi: 10.3382/ps.2008-00237 19151338

[14] Habig C, Geffers R, Distl O. Differential Gene Expression from Genome-Wide Microarray Analyses Distinguishes Lohmann Selected Leghorn and Lohmann Brown Layers. PLoS One. 2012;7(10). doi: 10.1371/journal.pone.0046787 23056453 PMC3466173

[15] Reyer H, Oster M, Ponsuksili S, Trakooljul N, Omotoso AO, Iqbal MA, et al. Transcriptional responses in jejunum of two layer chicken strains following variations in dietary calcium and phosphorus levels. BMC Genomics. 2021;22(1):1–12. 34187361 10.1186/s12864-021-07814-9 PMC8243909

[16] Sommerfeld V, Huber K, Bennewitz J, Camarinha-Silva A, Hasselmann M, Ponsuksili S, et al. Phytate degradation, myo-inositol release, and utilization of phosphorus and calcium by two strains of laying hens in five production periods. Poult Sci. 2020; 10.1016/j.psj.2020.08.064 PMC7704748 33248595

[17] Ponsuksili S, Hadlich F, Reyer H, Oster M, Trakooljul N, Iqbal MA, et al. Genetic background and production periods shape the microRNA profiles of the gut in laying hens. Genomics [Internet]. 2021;113(4):1790–801. Available from: doi: 10.1016/j.ygeno.2021.04.018 33848585

[18] Marchi S, Giorgi C, Suski JM, Agnoletto C, Bononi A, Bonora M, et al. Mitochondria-Ros Crosstalk in the Control of Cell Death and Aging. J Signal Transduct. 2012;2012:17. doi: 10.1155/2012/329635 22175013 PMC3235816

[19] Bose S, French S, Evans FJ, Joubert F, Balaban RS. Metabolic network control of oxidative phosphorylation. Multiple roles of inorganic phosphate. J Biol Chem [Internet]. 2003;278(40):39155–65. Available from: doi: 10.1074/jbc.M306409200 12871940

[20] Rizzuto R, De Stefani D, Raffaello A, Mammucari C. Mitochondria as sensors and regulators of calcium signalling. Nat Rev Mol Cell Biol [Internet]. 2012;13:566–78. Available from: doi: 10.1038/nrm3412 22850819

[21] Clapham DE. Calcium Signaling. Cell. 2007;131(6):1047–58. doi: 10.1016/j.cell.2007.11.028 18083096

[22] Dolmetsch RE, Xu K, Lewis RS. Calcium oscillations increase the efficiency and specificity of gene expression. Nature [Internet]. 1998 Apr 30 [cited 2021 May 17];392(6679):933–6. Available from: https://pubmed.ncbi.nlm.nih.gov/9582075/ doi: 10.1038/31960 9582075

[23] Gellerich FN, Gizatullina Z, Trumbeckaite S, Nguyen HP, Pallas T, Arandarcikaite O, et al. The regulation of OXPHOS by extramitochondrial calcium. Biochim Biophys Acta—Bioenerg. 2010;1797(6–7):1018–27. doi: 10.1016/j.bbabio.2010.02.005 20144582

[24] Loublier S, Bayot A, Rak M, El-Khoury R, Bénit P, Rustin P. The NDUFB6 subunit of the mitochondrial respiratory chain complex I is required for electron transfer activity: A proof of principle study on stable and controlled RNA interference in human cell lines. Biochem Biophys Res Commun. 2011 Oct 22;414(2):367–72. doi: 10.1016/j.bbrc.2011.09.078 21964293

[25] Bratic A, Larsson N. The role of mitochondria in aging. J Clin Invest. 2013;123(3):951–7. doi: 10.1172/JCI64125 23454757 PMC3582127

[26] Kokoszka JE, Coskun P, Esposito LA, Wallace DC. Increased mitochondrial oxidative stress in the Sod2 (+/-) mouse results in the age-related decline of mitochondrial function culminating in increased apoptosis. Proc Natl Acad Sci U S A. 2001;98(5):2278–83. doi: 10.1073/pnas.051627098 11226230 PMC30129

[27] Anderson R, Prolla T. PGC-1α in aging and anti-aging interventions. Biochim Biophys Acta—Gen Subj. 2009;1790(10):1059–66. 10.1016/j.bbagen.2009.04.005 PMC2743759 19371772

[28] Caverzasio J, Montessuit C, Bonjour JP. Renal Pi Transport and Plasma 1, 25-Dihydroxyvitamin. Endocrinology. 1990;127(1):453–9. 2361480 10.1210/endo-127-1-453

[29] Kasukawa Y, Baylink DJ, Wergedal JE, Amaar Y, Srivastava AK, Guo R, et al. Lack of Insulin-Like Growth Factor I Exaggerates the Effect of Calcium Deficiency on Bone Accretion in Mice. Endocrinology. 2003;144(11):4682–9. doi: 10.1210/en.2003-0745 12960002

[30] Bhattacharya TK, Chatterjee RN, Dushyanth K, Paswan C, Shukla R, Shanmugam M. Polymorphism and expression of insulin-like growth factor 1 (IGF1) gene and its association with growth traits in chicken. Br Poult Sci. 2015;56(4):398–407. doi: 10.1080/00071668.2015.1041098 26059224

[31] Heumann-Kiesler C, Sommerfeld V, Iffland H, Bennewitz J, Rodehutscord M, Hasselmann M. Insights into the Mitochondrial and Nuclear Genome Diversity of Two High Yielding Strains of Laying Hens. Animals. 2021;11(3):1–23. doi: 10.3390/ani11030825 33804055 PMC8001891

[32] Dreyling C, Hasselmann M. The dynamics of mitochondrial-linked gene expression among tissues and life stages in two contrasting strains of laying hens. PLoS One [Internet]. 2022;17(1):e0262613. Available from: doi: 10.1371/journal.pone.0262613 35025974 PMC8757906

[33] Andersen CL, Jensen JL, Ørntoft TF. Normalization of real-time quantitative reverse transcription-PCR data: A model-based variance estimation approach to identify genes suited for normalization, applied to bladder and colon cancer data sets. Cancer Res. 2004;64(15):5245–50. doi: 10.1158/0008-5472.CAN-04-0496 15289330

[34] Pfaffl MW. A new mathematical model for relative quantification in real-time RT-PCR. Nucleic Acids Res. 2001;29(9):e45. doi: 10.1093/nar/29.9.e45 11328886 PMC55695

[35] Vandesompele J, De Preter K, Pattyn F, Poppe B, Van Roy N, De Paepe A, et al. Accurate normalization of real-time quantitative RT-PCR data by geometric averaging of multiple internal control genes. Genome Biol. 2002;3. doi: 10.1186/gb-2002-3-7-research0034 12184808 PMC126239

[36] Hellemans J, Mortier G, De Paepe A, Speleman F, Vandesompele J. qBase relative quantification framework and software for management and automated analysis of real-time quantitative PCR data. Genome Biol. 2007;8(R19). doi: 10.1186/gb-2007-8-2-r19 17291332 PMC1852402

[37] Kuznetsova A, Brockhoff PB, Christensen RHB. lmerTest Package: Tests in Linear Mixed Effects Models. J Stat Softw. 2017;82(13).

[38] Lenth R, Singmann H, Love J, Buerkner P, Herve M. Package ‘emmeans’ [Internet]. R package version 1.4.6. 2020. Available from: https://cran.r-project.org/package=emmeans

[39] Sarle WS. Cubic clustering criterion. SAS Tech Report A -108. 1983.

[40] Ward JH. Hierarchical Grouping to Optimize an Objective Function. J Am Stat Assoc. 1963;58(301):236–44.

[41] Matalon O, Horovitz A, Levy ED. Different subunits belonging to the same protein complex often exhibit discordant expression levels and evolutionary properties. Curr Opin Struct Biol. 2014;26(1):113–20. 24997301 10.1016/j.sbi.2014.06.001

[42] De Lichtenberg U, Jensen LJ, Brunak S, Bork P. Dynamic complex formation during the yeast cell cycle. Science (80-). 2005;307(5710):724–7. doi: 10.1126/science.1105103 15692050

[43] van Waveren C, Moraes CT. Transcriptional co-expression and co-regulation of genes coding for components of the oxidative phosphorylation system. BMC Genomics. 2008;9(18). doi: 10.1186/1471-2164-9-18 18194548 PMC2268925

[44] Peterman EM, Sullivan C, Goody MF, Rodriguez-Nunez I, Yoder JA, Kim CH. Neutralization of mitochondrial superoxide by superoxide dismutase 2 promotes bacterial clearance and regulates phagocyte numbers in zebrafish. Infect Immun. 2015;83(1):430–40. doi: 10.1128/IAI.02245-14 25385799 PMC4288898

[45] Scott I. The role of mitochondria in the mammalian antiviral defense system. Mitochondrion [Internet]. 2010;10(4):316–20. Available from: doi: 10.1016/j.mito.2010.02.005 20206303 PMC2874622

[46] Ott M, Gogvadze V, Orrenius S, Zhivotovsky B. Mitochondria, oxidative stress and cell death. Apoptosis. 2007;12(5):913–22. doi: 10.1007/s10495-007-0756-2 17453160

[47] Holley AK, Dhar SK, Xu Y, Clair DKS. Manganese superoxide dismutase: Beyond life and death. Amino Acids. 2012;42(1):139–58. doi: 10.1007/s00726-010-0600-9 20454814 PMC2975048

[48] Santos AL, Sinha S, Lindner AB. The good, the bad, and the ugly of ROS: New insights on aging and aging-related diseases from eukaryotic and prokaryotic model organisms. Oxid Med Cell Longev. 2018;2018. 10.1155/2018/1941285 PMC5878877 29743972

[49] Yin. W, Robyn. B, Alycia. N, Siegfired. H. Superoxide dismutases: Dual roles in controlling ROS damage and regulating ROS signaling. J Cell Biol. 2018;217(6):1915–28. doi: 10.1083/jcb.201708007 29669742 PMC5987716

[50] Blake LE, Roux J, Hernando-Herraez I, Banovich NE, Perez RG, Hsiao CJ, et al. A comparison of gene expression and DNA methylation patterns across tissues and species. Genome Res. 2020;30(2):250–62. doi: 10.1101/gr.254904.119 31953346 PMC7050529

[51] Campbell NA, Reece JB. Biology. 8th ed. San Francisco: Pearson Education, Inc.; 2008.

[52] Nicholson JK, Holmes E, Kinross J, Burcelin R, Gibson G, Jia W, et al. Metabolic Interactions. Science (80-). 2012;108(June):1262–8. 10.1126/science.1223813 22674330

[53] Saltiel AR, Kahn CR. Insulin signalling and the regulation of glucose and lipid metabolism. Nature [Internet]. 2001 Dec 13 [cited 2021 Apr 26];414:799–806. Available from: http://www.medgen.med.umich.edu/labs/saltiel/. doi: 10.1038/414799a 11742412

[54] Broughton S, Partridge L. Insulin/IGF-like signalling, the central nervous system and aging. Biochem J. 2009;418(1):1–12. doi: 10.1042/BJ20082102 19159343

[55] Brogiolo W, Stocker H, Ikeya T, Rintelen F, Fernandez R, Hafen E. An evolutionarily conserved function of the drosophila insulin receptor and insulin-like peptides in growth control. Curr Biol [Internet]. 2001 Feb 20 [cited 2021 Apr 26];11(4):213–21. Available from: https://pubmed.ncbi.nlm.nih.gov/11250149/ doi: 10.1016/s0960-9822(01)00068-9 11250149

[56] Butler AA, Le Roith D. Control of growth by the somatropic axis: Growth hormone and the insulin-like growth factors have related and independent roles. Annu Rev Physiol [Internet]. 2001 [cited 2021 Apr 26];63:141–64. Available from: https://pubmed.ncbi.nlm.nih.gov/11181952/ doi: 10.1146/annurev.physiol.63.1.141 11181952

[57] Savage MO. Insulin-like growth factors, nutrition and growth. World Rev Nutr Diet. 2013;106:52–9. doi: 10.1159/000342577 23428681

[58] Pedroso FL, De Jesus-Ayson EGT, Cortado HH, Hyodo S, Ayson FG. Changes in mRNA expression of grouper (Epinephelus coioides) growth hormone and insulin-like growth factor I in response to nutritional status. Gen Comp Endocrinol [Internet]. 2006 [cited 2021 Apr 26];145(3):237–46. Available from: https://pubmed.ncbi.nlm.nih.gov/16243324/ doi: 10.1016/j.ygcen.2005.09.001 16243324

[59] Calikoglu AS, Karayal AF, D’Ercole AJ. Nutritional regulation of IGF-I expression during brain development in mice. Pediatr Res [Internet]. 2001 [cited 2021 Apr 26];49(2):197–202. Available from: https://pubmed.ncbi.nlm.nih.gov/11158513/ doi: 10.1203/00006450-200102000-00011 11158513

[60] Fox BK, Riley LG, Hirano T, Grau EG. Effects of fasting on growth hormone, growth hormone receptor, and insulin-like growth factor-I axis in seawater-acclimated tilapia, Oreochromis mossambicus. Gen Comp Endocrinol [Internet]. 2006 Sep 15 [cited 2021 Apr 26];148(3):340–7. Available from: https://pubmed.ncbi.nlm.nih.gov/16750210/ doi: 10.1016/j.ygcen.2006.04.007 16750210

[61] Onagbesan OM, Vleugels B, Buys N, Bruggeman V, Safi M, Decuypere E. Insulin-like growth factors in the regulation of avian ovarian functions. Domest Anim Endocrinol. 1999;17(2–3):299–313. doi: 10.1016/s0739-7240(99)00046-6 10527132

