# Supplementary material for: The impact of dietary calcium and phosphorus on mitochondrial-linked gene expression in five tissues of laying hens
Source: PLoS One. 2022 Jun 24;17(6):e0270550. doi: 10.1371/journal.pone.0270550 (PMC9231785; doi:10.1371/journal.pone.0270550)
Supplement: S1 File — (DOCX) [file pone.0270550.s001.docx]

**Table A:** Primer sequences for 35 genes that were used in this study with product size, primer efficiency and accession number of the reference sequence that was used to design the primers. The primer of *GAPDH* is already published in Hong *et al.* 2012, all primers except *ATP50*, *ND2*, *ND3*, *PRKAB1*, and *PRKAG3* are already published in Heumann-Kiesler *et al.* 2021.

| Gene | Forward | Reverse | Product-size [bp] | Efficiency | Accession  Number |  |
| --- | --- | --- | --- | --- | --- | --- |
| *ACTB* | GCTGTGCTGTCCCTGTATGC | TTCTCTCTCGGCTGTGGTGG | 210 | 95.08 | NM_205518 |  |
| *ATP6* | CCGATGGATCAACAACCGCC | AGCTGGGTAGTTGGGGTGAA | 189 | 98.26 | AP003317 |  |
| *ATP50* | CTGTGGGAGAAAATGGCGGC | CCCAGCTCCTTCTCCACCTG | 191 | 94.23 | XM_416717 |  |
| *ATP5F1* | GCCAGAGAAGGGAGGAGAGG | TGACGCCGGTTTTGGGATAGA | 82 | 106.93 | NC_006113 |  |
| *ATP8* | GCCCCAATTAAACCCAAACCCA | TGCAGGGTTGTTTGTTAGAGTGA | 112 | 105.34 | AP003317 |  |
| *COXC6* | CCTTTTGGCCAGGCGGATGA | TTGGGCGGTGCAGACTCAAA | 192 | 96.93 | NT_455816 |  |
| *COX1* | AGCCTTCTAATCCGCGCAGA | AGGAGGGAGGGAGGAGTCAG | 226 | 94.72 | AP003317 |  |
| *COX2* | GACCACGCCCTGATAGTCGC | GGGCAAGCAGGACTAGGACA | 157 | 102.28 | AP003317 |  |
| *COX3* | CCACTACAGCTCGACCACCC | GGTGTTGGGGCTAGGCTTGA | 225 | 107.68 | AP003317 |  |
| *COX5A* | CCACGGGTCACAGGAGTCAG | TGAAATACGTCACCCAGCGG | 56 | 95.17 | NC_006097 |  |
| *CytB* | CCCAGCCCCATCCAACATCT | AGGCCTCGTCCGATGTGAAG | 243 | 88.2 | AP003317 |  |
| *GAPDH* | TGCTGCCCAGAACATCATCC | ACGGCAGGTCAGGTCAACAA | 142 | 103.12 | K01458 | |
| *IGF-1α* | TGGCCTGTGTTTGCTTACCTTA | AGCCTCTGTCTCCACATACGA | 107 | 92.61 | JN_593011-18 | |
| *MTOR* | GGCACTGTGTCTATTCTCCAACA | GCAGGTCCTTGGCAGCTTTC | 85 | 93.98 | XM_417614.5 | |
| *ND1* | TCCCCATCCTAATCGCCGTG | GGGAATGGTAGTGGGAGGGG | 250 | 95.42 | AP003317 | |
| *ND2* | AACCAACGCCTGATCCACCG | GGCAGTGATTAGGGAGGAGCC | 157 | 95.42 | AP003317 | |
| *ND3* | GGATCAGCCCGACTCCCATT | GGGCGATGATAGTGGTGGCT | 158 | 97.39 | AP003317 | |
| *ND4* | CACCCACCCCAACCTACCTG | AGGGTGAGGAAGGGGTAGTGT | 249 | 95.60 | AP003317 | |
| *ND4L* | GTCTCCCCTACACTTCAGCTTCT | GGGCGAATGATGGGGTTTGG | 170 | 91.09 | AP003317 | |
| *ND5* | CCCACCCAAACCAAACACCC | CATAGGCACGTTGTCAGGGC | 229 | 89.91 | AP003317 | |
| *ND6* | TCAACGAGCCCTCCCAGAAC | GGGTTGGTGGTAGCGTCTGT | 236 | 98.57 | AP003317 | |
| *NDUFB6* | CTGATCCCTGCCTGGCTCC | GCCTTCTTTGCTGCAGGACG | 208 | 94.99 | NC_006101 | |
| *PGC1α* | ACAAAAGCCACAAAGACGTCCCT | GCTGCTGTTCCTGTTCTCTGCT | 100 | 100.69 | NC_006091 | |
| *PPIA* | TGACTTTACGCGCCACAACG | TCGGTCTTGGCAGTGCAGAT | 165 | 93.55 | NC_006109.5 | |
| *PRKAA1* | GCGGCAGATAAACAGAAGCACG | CGTGTCGCCCAGAATGTAATGC | 66 | 97.63 | NC_006127 | |
| *PRKAA2* | TGACGGGGCACAAAGTAGCA | TGAGGGTGCCGGAAGAGTTT | 115 | 96.51 | NC_006095 | |
| *PRKAB1* | ATGGGGAACACGAGCAGC | TCGGTCTGTCCCCATCCTTG | 109 | 99.75 | NC_006102 | |
| *PRKAB2* | GAACACCACCAGCGAGCGAG | ACTGGGGTCATCTGTGCTGC | 133 | 89.7 | NC_006095 | |
| *PRKAG2* | AGCAGCCCACGTTTCCTCTT | GCCGCTGGTTTTGAGGTAGC | 175 | 105.29 | XM_015281206-9 | |
| *PRKAG3* | CAAGCACAAGATCCACCGCC | TTCTCAGGTACGACGGCCAC | 192 | 97.99 | XM_015289796-7 | |
| *SDHA* | TGTTGTGGGTGCAGGAGGAG | TGGGGCTTGCTCAGTCATGT | 247 | 96.03 | NT_455790 | |
| *SDHB* | TCGAGGAGCTCAGACGGC | CCCCAGGCTTATCAGGATCCC | 83 | 95.03 | NC_006108 | |
| *SOD2* | ACACTCTTCCTGACCTGCCTT | CCTTTTGCCAGCGCCTCTTT | 151 | 95.81 | NC_006090 | |
| *UQCRC1* | CCCTGCTGCCTCTGACGA | GTTGGCTGGCTGGACTCCTC | 141 | 103.96 | NC_006099 | |
| *UQCRC2* | GTAGCGCCGAAAGTTGCAGT | CGCCCGGACACAGCTTCA | 58 | 91.14 | NC_006101 | |

**Table B:** PCR conditions used for all PCR reactions during evaluation of specificity, using Dream Taq (Thermo Fisher scientific Inc., Massachusetts, USA).

| Temperature [°C] | Time |  |
| --- | --- | --- |
| 95 | 2 min |  |
| 95 | 30 s | 30× |
| 60 | 30 s |  |
| 72 | 1 min |  |
| 72 | 5 min |  |
| 4 | ∞ |  |

**Table C:** Thermal cycling parameters used on the final qPCR runs according to the manufacturers protocol (Fluidigm Corporation, San Francisco, USA).

| Step | Flex Six | | 96×96 | |
| --- | --- | --- | --- | --- |
|  | Temperature [°C] | duration | Temperature [°C] | duration |
| Thermal mixing | 25 | 30 min | 70 | 40 min |
|  | 70 | 60 min | 60 | 30 sec |
| Hot start | 95 | 1 min | 95 | 1 min |
| PCR, 30 cycles | 96 | 5 sec | 96 | 5 sec |
|  | 60 | 20 sec | 60 | 20 sec |
| Melting curve | 60 | 3 sec | 60 | 3 sec |
|  | 60-95 | 1°C/3sec | 60-95 | 1°C/3sec |

**Table D:** Relative gene expression of *NDUFB6* and *SOD2* in breast muscle tissue. Shown emmeans and standard errors derived from the statistical model. Tukey HSD tests were used to test for significance.

| gene | emmean± SE | emmean± SE | p-value |
| --- | --- | --- | --- |
|  | Diet 1 (P+Ca+) | Diet 4 (P-Ca+) |  |
| *SOD2* | 4.7798±0.16 | 4.2210±0.16 | 0.0195 |
| *NDUFB6* | 4.421±0.328 | 3.159±0.322 | 0.0059 |
|  | Diet 2 (P-Ca+) | Diet 4 (P-Ca+) |  |
| *SOD* | 4.717±0.16 | 4.2210±0.16 | 0.0488 |
|  | Diet 3 (P+Ca-) | Diet 4 (P-Ca+) |  |
| *NDUFB6* | 4.167±0.328 | 3.159±0.322 | 0.0432 |
