# Supplementary material for: The impact of dietary calcium and phosphorus on mitochondrial-linked gene expression in five tissues of laying hens
Source: PLoS One. 2022 Jun 24;17(6):e0270550. doi: 10.1371/journal.pone.0270550 (PMC9231785; doi:10.1371/journal.pone.0270550)
Supplement: S1 Table — p-values from the three-factorial anova obtained from the linear mixed model. Statistical significance was declared when p < 0.05. (DOCX) [file pone.0270550.s004.docx]

**Table S1:** **Significant influence of strain, diet, tissue and all possible interactions on gene expression per gene.** p-values from the three-factorial anova obtained from the linear mixed model. Statistical significance was declared when p < 0.05.

| Gene | strain | diet | tissue | Tissue:diet | Strain:tissue | Strain:diet | Strain:tissue:diet |
| --- | --- | --- | --- | --- | --- | --- | --- |
| *ATP6* | ns | ns | <2.2E-16 | ns | 0.02742 | ns | ns |
| *ATP5F1* | ns | ns | <2.2E-16 | ns | ns | ns | ns |
| *ATPF0* | ns | ns | <2.2E-16 | ns | ns | ns | ns |
| *ATP8* | ns | ns | <2.2E-16 | ns | ns | ns | ns |
| *COXC6* | ns | ns | <2.2E-16 | ns | ns | ns | ns |
| *COX1* | ns | ns | <2.2E-16 | ns | ns | ns | ns |
| *COX2* | ns | ns | <2.2E-16 | ns | ns | ns | ns |
| *COX3* | ns | ns | <2.2E-16 | ns | 0.04962 | ns | ns |
| *COX5A* | ns | ns | <2.2E-16 | ns | ns | ns | ns |
| *CytB* | ns | ns | <2.2E-16 | ns | 0.01515 | ns | ns |
| *GAPDH* | ns | ns | <2.2E-16 | ns | ns | ns | ns |
| *IGF-1α* | ns | ns | <2.2E-16 | ns | <2E-16 | ns | 0.028 |
| *MTOR* | ns | ns | <2.2E-16 | ns | ns | ns | ns |
| *ND1* | ns | ns | <2.2E-16 | ns | ns | ns | ns |
| *ND2* | ns | ns | <2.2E-16 | ns | ns | ns | ns |
| *ND3* | ns | ns | <2.2E-16 | ns | 0.02034 | 0.02989 | ns |
| *ND4* | ns | ns | <2.2E-16 | ns | ns | ns | ns |
| *ND4L* | ns | ns | <2.2E-16 | ns | ns | ns | ns |
| *ND5* | ns | ns | <2.2E-16 | ns | 0.01722 | ns | ns |
| *ND6* | ns | ns | <2.2E-16 | ns | ns | ns | ns |
| *NDUFB6* | ns | 0.04104 | <2.2E-16 | ns | ns | ns | ns |
| *PGC1α* | ns | ns | <2.2E-16 | ns | ns | ns | ns |
| *PRKAA1* | ns | ns | <2.2E-16 | ns | ns | ns | ns |
| *PRKAA2* | ns | ns | <2.2E-16 | ns | ns | ns | ns |
| *PRKAB1* | ns | ns | <2.2E-16 | ns | ns | ns | ns |
| *PRKAB2* | ns | ns | <2.2E-16 | ns | 0.005763 | ns | ns |
| *PRKAG2* | ns | ns | <2.2E-16 | ns | ns | ns | ns |
| *PRKAG3* | ns | ns | <2.2E-16 | ns | ns | ns | ns |
| *SDHA* | ns | ns | <2.2E-16 | ns | ns | ns | ns |
| *SDHB* | ns | ns | <2.2E-16 | ns | ns | ns | ns |
| *SOD2* | ns | 0.008355 | <2.2E-16 | ns | ns | ns | ns |
| *UQCRC1* | ns | ns | <2.2E-16 | ns | ns | ns | ns |
| *UQCRC2* | ns | ns | <2.2E-16 | ns | ns | ns | ns |
