# Supplementary material for: The impact of dietary calcium and phosphorus on mitochondrial-linked gene expression in five tissues of laying hens
Source: PLoS One. 2022 Jun 24;17(6):e0270550. doi: 10.1371/journal.pone.0270550 (PMC9231785; doi:10.1371/journal.pone.0270550)
Supplement: S2 Table — (DOCX) [file pone.0270550.s005.docx]

**Table S2. Number of samples per gene and tissue after the removal of outliers used to calculate emmeans from the statistical model.**

| Gene/tissue | breast | duodenum | ileum | liver | ovary |
| --- | --- | --- | --- | --- | --- |
| ***ATP6*** | 80 | 80 | 80 | 80 | 79 |
| ***ATP8*** | 80 | 80 | 80 | 80 | 80 |
| ***COX1*** | 80 | 80 | 79 | 80 | 75 |
| ***COX2*** | 80 | 80 | 80 | 80 | 79 |
| ***COX3*** | 80 | 80 | 80 | 79 | 79 |
| ***ND1*** | 80 | 80 | 79 | 79 | 77 |
| ***ND2*** | 80 | 80 | 80 | 80 | 80 |
| ***ND3*** | 80 | 80 | 80 | 80 | 80 |
| ***ND4*** | 80 | 80 | 80 | 80 | 76 |
| ***ND4L*** | 80 | 80 | 79 | 79 | 79 |
| ***ND5*** | 55 | 58 | 58 | 58 | 46 |
| ***ND6*** | 77 | 79 | 79 | 75 | 73 |
| ***ATPF0*** | 69 | 69 | 67 | 60 | 62 |
| ***ATP5F1*** | 80 | 80 | 80 | 80 | 80 |
| ***COX5A*** | 80 | 80 | 80 | 80 | 80 |
| ***COXC6*** | 79 | 78 | 78 | 80 | 75 |
| ***CytB*** | 80 | 80 | 80 | 80 | 79 |
| ***NDUFB6*** | 78 | 79 | 79 | 79 | 74 |
| ***UQCRC1*** | 74 | 80 | 80 | 80 | 80 |
| ***UQCRC2*** | 80 | 80 | 80 | 80 | 80 |
| ***SDHA*** | 79 | 79 | 78 | 76 | 74 |
| ***SDHB*** | 80 | 80 | 80 | 80 | 80 |
| ***IGF-1*** | 76 | 79 | 79 | 75 | 72 |
| ***MTOR*** | 78 | 79 | 80 | 80 | 80 |
| ***PRKAA1*** | 79 | 79 | 80 | 78 | 79 |
| ***PRKAA2*** | 80 | 76 | 77 | 66 | 65 |
| ***PRKAB1*** | 65 | 61 | 71 | 51 | 69 |
| ***PRKAB2*** | 80 | 80 | 80 | 79 | 79 |
| ***PRKAG2*** | 70 | 75 | 78 | 79 | 78 |
| ***PRKAG3*** | 80 | 56 | 58 | 54 | 51 |
| ***SOD2*** | 80 | 80 | 80 | 80 | 77 |
| ***PGC1-a*** | 72 | 79 | 79 | 79 | 75 |
| ***GAPDH*** | 80 | 80 | 80 | 80 | 80 |
